# Reply to P. Boffetta and M. Seyyedsalehi

**DOI:** 10.1200/GO-24-00460

**Published:** 2024-10-24

**Authors:** Thilagavathi Ramamoorthy, Anita Nath, Shubhra Singh, Stany Mathew, Apourv Pant, Samvedana Sheela, Gurpreet Kaur, Krishnan Sathishkumar, Prashant Mathur

**Affiliations:** Thilagavathi Ramamoorthy, PhD, Indian Council of Medical Research—National Centre for Disease Informatics and Research, Bengaluru, India; Anita Nath, MD, Indian Council of Medical Research—National Centre for Disease Informatics and Research, Bengaluru, India; Shubhra Singh, PhD, Indian Council of Medical Research—National Centre for Disease Informatics and Research, Bengaluru, India; Stany Mathew, MPH, Indian Council of Medical Research—National Centre for Disease Informatics and Research, Bengaluru, India; Apourv Pant, PhD, Indian Council of Medical Research—National Centre for Disease Informatics and Research, Bengaluru, India; Samvedana Sheela, MD, Indian Council of Medical Research—National Centre for Disease Informatics and Research, Bengaluru, India; Gurpreet Kaur, MSc, Indian Council of Medical Research—National Centre for Disease Informatics and Research, Bengaluru, India; Krishnan Sathishkumar, MSc, Indian Council of Medical Research—National Centre for Disease Informatics and Research, Bengaluru, India; Prashant Mathur, PhD, DNB, Indian Council of Medical Research—National Centre for Disease Informatics and Research, Bengaluru, India

Thank you for the opportunity to respond to the recent feedback from Boffetta et al on our wok “Assessing the Global Impact of Ambient Air Pollution on Cancer Incidence and Mortality: A Comprehensive Meta-Analysis.” We appreciate the engagement with our work and welcome the discussion it has generated.

The authors are concerned about combining risk estimates for different pollutants (PM_2.5_, PM_10_, NO_2_, and O_3_) and different type of cancers could lead to biased results because of the differing impacts of these pollutants on cancer risk. Keeping this in mind, we had conducted subgroup analyses to explore the associations between specific pollutants and specific cancers. These analyses are detailed in table 1 and supplementary figures 3-5 of our manuscript. Moreover, the pooled risk estimates of subgroup analyses (by site of cancer and pollutants) are within the confidence limits of combined risk estimates, validating the results of our study. The focus of this study was not on specific histologic types of cancers, and data on this were limited in the included studies. We plan to address this aspect in our future work.

Regarding the concern about rescaling results to a common exposure contrast, we clarify that we pooled relative risks using a standardized increment of pollutant concentration: 10 μg/m^3^ for PM_2.5_ and PM_10_ and 10 parts per billion for NO_2_ and ozone. This method aligns with the standard practices used in other studies and cited in our manuscript.^1-3^ The standardization was performed as outlined in the statistical analysis section, ensuring consistency in risk estimates across studies.

The inclusion of multiple risk estimates from the same study does not exaggerate the results. We ensured that each estimate was distinct and specifically related to the association between a particular air pollutant and a specific cancer type. For instance, studies such as those by Gandini et al^4^ and Turner et al^5^ examined multiple exposures and cancer outcomes, and each estimate was treated separately and appropriately in our meta-analysis using the random-effects model addressing heterogeneity in the population and pollutants. This approach accounts for variations across studies, providing more reliable and generalized estimates while mitigating concerns about the validity of our findings.

Regarding the potential bias from studies that did not adjust for confounders, we included only adjusted risk estimates in our meta-analysis to statistically account for the influence of confounding factors, as detailed in the Data Sources and Search Strategy section of our manuscript. In addition, we assessed the impact of confounding factors in the Risk of Bias section, which is presented in the supplementary tables 4 and 5. We also discussed the potential for bias in our discussion, noting that most studies on cancer incidence were assessed to have a low risk of bias, which helps to minimize the influence of such factors on our overall conclusions.
